# Non-invasive brain stimulation contributing to postural control with and without stroke: a systematic review and meta-analysis

**DOI:** 10.1038/s41598-025-07840-7

**Published:** 2025-07-18

**Authors:** Sangha Cha, Jayoung Choi, Changwon Moon, Kanghee Cho

**Affiliations:** 1 Department of Rehabilitation Medicine, Chungnam National University College of Medicine, Daejeon, Republic of Korea; 2 Department of Biomedical Institute, Chungnam National University, Daejeon, Republic of Korea

**Keywords:** Brain stimulation, Transcranial direct current stimulation, Transcranial magnetic stimulation, Postural balance, Stroke, Health care, Medical research, Neurology

## Abstract

Non-invasive brain stimulation techniques demonstrate promising potential for enhancing neural plasticity and motor recovery, yet their comparative effectiveness for improving postural control across neurologically impaired and intact populations requires systematic investigation. This systematic review and meta-analysis evaluated the therapeutic efficacy of brain stimulation modalities on postural control, comparing outcomes between stroke survivors and neurologically intact adults through controlled trials. Systematic searches were conducted across major databases (CINAHL, Embase, MEDLINE, Web of Science) for randomized controlled trials and crossover studies published from 2014 to 2024. Study quality was assessed using the Risk of Bias 2 tool, with treatment effects analyzed through standardized mean differences in a random-effects model. Analysis of 15 studies revealed significant overall effects of brain stimulation (SMD = 0.79, 95% CI 0.47–1.11), with notably stronger responses in stroke participants (SMD = 0.95) versus neurologically intact individuals (SMD = 0.39). Transcranial direct current stimulation showed particular efficacy in stroke rehabilitation (SMD = 1.79), while intermittent theta burst stimulation demonstrated moderate effects (SMD = 0.68). Primary motor cortex stimulation yielded optimal outcomes (SMD = 1.21), followed by cerebellar (SMD = 0.75) and dorsolateral prefrontal cortex interventions (SMD = 0.35). These findings reveal differential response patterns between populations and stimulation parameters, suggesting enhanced neuroplastic potential in stroke survivors. This evidence supports the development of targeted neuromodulatory approaches for rehabilitation and performance enhancement.

## Introduction

The global healthcare landscape continues to grapple with stroke as a leading cause of mortality and long-term disability. Clinical evidence indicates that approximately two-thirds of stroke survivors experience persistent challenges with motor function and postural stability^1^. The pathophysiological cascade following stroke particularly impacts cortical and subcortical regions, disrupting the intricate neural networks essential for motor control. This neural disruption manifests prominently in compromised postural mechanisms^2^, fundamentally affecting individuals’ capacity to maintain balance—a cornerstone of independent living and fall prevention^3^.

The brain’s remarkable capacity for neuroplasticity has emerged as a central focus in rehabilitation science, offering promising pathways for functional recovery through neural reorganization and adaptation^4^. Within this context, Non-Invasive Brain Stimulation (NIBS) techniques have emerged as pioneering therapeutic tools. The NIBS spectrum encompasses various modalities, including transcranial direct current stimulation (tDCS), transcranial magnetic stimulation (TMS), repetitive TMS (rTMS), transcranial alternating current stimulation (tACS), paired associative stimulation (PAS), and theta burst stimulation (TBS). These approaches share the fundamental capability to modulate cortical excitability with remarkable precision^5,6^.

Intriguingly, the neurophysiological response to NIBS demonstrates distinct patterns between stroke survivors and neurologically intact individuals^7^. In the post-stroke brain, NIBS facilitates neural recovery through dual mechanisms: reinvigorating compromised neural pathways while simultaneously strengthening compensatory circuits^8^. Conversely, in healthy individuals, NIBS appears to enhance existing neural efficiency, optimizing synaptic transmission and neural network performance^9,10^. The intact postural control system relies on sophisticated integration of visual, vestibular, and somatosensory inputs^11^, with NIBS showing potential to enhance this complex sensory processing^12^.

The scientific literature documents encouraging outcomes in both populations. In stroke rehabilitation, NIBS has demonstrated capacity to enhance postural recovery^13^, while studies in healthy individuals reveal improvements in postural stability and dynamic balance performance^14,15^. These parallel yet distinct outcomes suggest that NIBS effects are intrinsically linked to the underlying neural state, carrying significant implications for therapeutic applications^16^.

Despite these advances, a comprehensive comparative analysis of NIBS effects on postural control between stroke survivors and neurologically intact individuals remains notably absent from the literature. For clarity, in this review, we define "neurologically intact individuals" as those without central nervous system disorders, distinguishing them from stroke survivors who experience neural disruption directly affecting central postural control mechanisms. While we acknowledge that some neurologically intact individuals may experience peripheral injuries affecting postural stability, the underlying neural processing of postural control remains fundamentally different from those with central nervous system lesions. This distinction is crucial for understanding the differential mechanistic response to NIBS between these populations. Such an investigation could illuminate the distinct neurophysiological responses to NIBS between individuals with and without central neurological damage, potentially guiding the development of more targeted and effective therapeutic protocols. This systematic review and meta-analysis aims to bridge this knowledge gap by quantitatively examining the differential effects of NIBS on postural control across both populations, providing evidence-based insights for clinical application.

## Results

### Study selection

Our systematic database search identified a total of 179 records. For studies without stroke condition, 36 records were identified (CINAHL: n = 13, Embase: n = 10, MEDLINE: n = 1, Web of Science: n = 12), while 156 records were found for studies with stroke condition (CINAHL: n = 19, Embase: n = 43, MEDLINE: n = 24, Web of Science: n = 70).

After removing duplicate records (n = 4 for non-stroke studies; n = 56 for stroke studies), 32 and 100 records remained for initial screening, respectively. Through the screening process, 26 non-stroke records and 71 stroke records were excluded based on title and abstract review. This yielded 6 non-stroke and 29 stroke-related articles for full-text assessment.

During the full-text review phase, articles were excluded based on various criteria. For non-stroke studies, one article was excluded due to insufficient data. For stroke studies, exclusions were made based on intervention type (n = 2), study design (n = 5), outcome measures (n = 11), and insufficient data (n = 1).

**Fig. 1:**
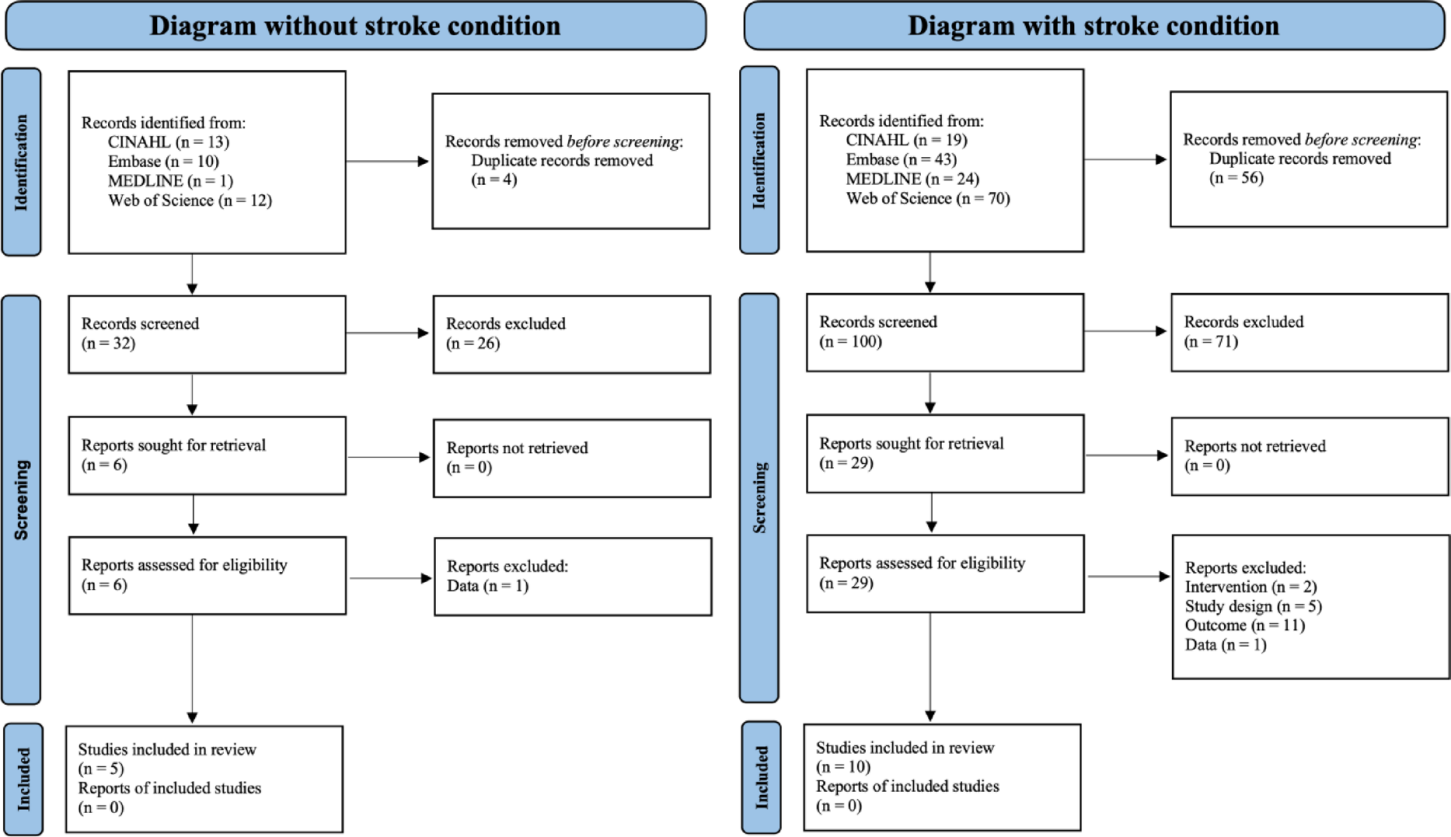
PRISMA flow diagram.

The final analysis included 15 studies meeting all eligibility criteria: 5 studies investigating NIBS effects in neurologically intact individuals^17–21^ and 10 studies examining NIBS applications in stroke patients^22–31^. No additional reports of included studies were identified for either group (Fig. 1).

This selection process followed the PRISMA 2020 guidelines, with independent assessment conducted by two researchers and any disagreements resolved through consensus.

### Study characteristics

This systematic review analyzed interventional characteristics of 15 studies, divided into non-stroke (n = 5) and stroke (n = 10) populations. Non-stroke studies primarily applied tDCS interventions over M1 and DLPFC regions. Intervention periods spanned from single sessions to multiple sessions (range: 5–10) over several weeks, primarily employing 2 mA stimulation intensity. Balance assessment methods included objective measures via force plates and standardized clinical tests.

Stroke-focused investigations employed diverse NIBS modalities targeting M1, cerebellar, and prefrontal regions. Cerebellar protocols predominantly utilized iTBS (80% AMT, 600–1200 pulses), while tDCS applications maintained consistent parameters (2 mA, 20-min sessions) across 2–4 week periods. Balance assessment primarily employed standardized clinical measures, with BBS being the most frequently utilized tool.

Demographics showed distinct age distributions between groups. Non-stroke participants represented younger populations (mean age range: 19–33 years), while stroke participants were typically older (mean age range: 51–69 years). Control conditions universally employed sham stimulation matching experimental parameters. Most investigations (86.7%) incorporated conventional rehabilitation or specific exercise protocols alongside NIBS applications.

The predominant study design featured multiple intervention sessions (mean duration: 2–4 weeks), with only two studies examining single-session effects. Despite methodological variations between studies, stimulation parameters maintained relative consistency within each NIBS modality category (Table 1).

**Table 1:** Characteristics of the included randomized controlled trials.

Study	Experimental group (age / intervention)	Control group (age / intervention)	Intervention intensity	Region of interest	Types of NIBS	Postural balance tools	Participants’ condition (Age of EG/CG)	Authors’ conclusion
Tohidirad et al. (2023)	Active M1 anodal tDCS + PT	Sham M1 tDCS + PT	2 mA, 20 min, 10 sessions over 2 weeks	M1	tDCS	COP with force plate	Athletes with partial ACL injury (31.53 ± 7.20/31.73 ± 7.05)	M1 a-tDCS + physical therapy improved postural control in ACL-injured athletes
Chaturvedi et al. (2022)	Active tDCS on DLPFC (anode) and Cz (cathode)	Sham tDCS	2 mA, 20 min, 5 sessions over 5 days	DLPFC	tDCS	Y-balance test	Athletes with ankle sprain (19.70 ± 3.06/22.80 ± 5.71)	tDCS improved pain and range of motion but not balance in ankle sprains
Jamebozorgi et al. (2023)	tDCS + ICE	Sham tDCS + ICE	1 mA, 20 min, 10 sessions over 4 weeks	Cerebellum	tDCS	SEBT; anterior	Athletes with ACL injury (29.91 ± 8.75/30.25 ± 1.25)	tDCS and biofeedback with exercises showed similar effects on functional balance
Song and Yim (2023)	tDCS + PCE	Sham tDCS + PCE	2 mA, 20 min, 8 sessions over 4 weeks	M1	tDCS	Y-balance test	Healthy adults (33.06 ± 4.60/33.26 ± 4.80)	Plyometric exercises + tDCS enhanced balance and body functions
Kaminski et al. (2016)	Anodal tDCS + DBT	Sham tDCS + DBT	1 mA, 20 min, 1 session	M1	tDCS	TiB	Healthy young adults (26.08 ± 3.19)	Anodal tDCS over M1 leg area facilitated dynamic balance learning in young adults
Zhu et al. (2024)	Cerebellar iTBS + standard PT	Sham iTBS + standard PT	80% AMT, 1200 pulses, 10 sessions over 2 weeks	Cerebellum	iTBS	BBS	First unilateral stroke, > 2 weeks post-stroke, lower limb deficits (58.67 ± 7.24/62.33 ± 8.78)	Cerebellar iTBS with physical therapy improved balance and gait in post-stroke hemiplegia
Xie et al. (2021)	Cerebellar iTBS + conventional PT	Sham iTBS + same conventional PT	80% AMT, 600 pulses, 10 sessions over 10 days	Cerebellum	iTBS	TUG	First unilateral stroke, < 6 months post-stroke (52.35 ± 8.62/54.41 ± 7.01)	Contralesional cerebellar iTBS enhanced walking performance
Liao et al. (2021)	Cerebellar iTBS + PT	Sham iTBS + PT (50 min daily)	80% AMT, 600 pulses, 10 sessions over 2 weeks	Cerebellum	iTBS	BBS	Subacute/chronic stroke, > 2 weeks post-stroke (51.53 ± 9.22/55.40 ± 8.10)	Cerebellar iTBS promoted balance and trunk function recovery
Koch et al. (2019)	CRB-iTBS + PT	Sham iTBS + PT	80% RMT, 1200 pulses, daily sessions over 3 weeks	Cerebellum	iTBS	BBS	First chronic ischemic stroke, ≥ 6 months post-stroke with hemiparesis (63 ± 11/65 ± 12)	Cerebellar iTBS improved motor relearning and cortical reorganization
Shah et al. (2021)	Anodal tDCS + exercises	Sham tDCS + exercises	2 mA, 20 min, 12 sessions over 3 weeks	M1	tDCS	BBS	Single stroke, MMSE ≥ 24, BBS 21–40	Both cathodal and anodal tDCS improved balance, with anodal showing superior results
	Cathodal tDCS + exercises		2 mA, 20 min, 12 sessions over 3 weeks					
Andrade et al. (2017)	Anodal tDCS + PT exercises	Sham tDCS + PT exercises	2 mA, 20 min, 10 sessions over 2 weeks	M1	tDCS	OSI	Acute ischemic stroke, independent 10 m walk (68.86 ± 4.66/68.00 ± 1.46)	Bilateral tDCS was more effective than unilateral for balance improvement
	Bilateral tDCS + PT exercises		2 mA, 20 min, 10 sessions over 2 weeks					
	Cathodal tDCS + PT exercises		2 mA, 20 min, 10 sessions over 2 weeks					
Yu et al. (2022)	rTMS on left DLPFC + RRT	Sham stimulation + RRT	5 Hz, 80% MT, 1200 pulses, 10 sessions over 2 weeks	DLPFC	rTMS	BBS	Acute stroke (< 3 months), basal ganglia, 10 m walk capable (54.6 ± 11.83/57.37 ± 12.78)	Left DLPFC rTMS improved executive function and postural control
Liao et al. (2024)	M1-iTBS + PT	Sham-iTBS + PT	80% RMT, 1200 pulses, 15 sessions over 3 weeks	M1	iTBS	BBS	Subacute first stroke (> 2 weeks), FMA-LE < 34, BBS < 56 (58.25 ± 14.63/57.08 ± 11.28)	Cerebellar-iTBS showed better motor recovery compared to M1-iTBS
	Cerebellar-iTBS + PT		80% RMT, 1200 pulses, 15 sessions over 3 weeks	Cerebellum				
Seo et al. (2017)	RAGT + anodal tDCS	RAGT + sham tDCS	2 mA, 20 min, 10 sessions over 2 weeks	M1	tDCS	BBS	Chronic stroke (> 6 months), FAC ≤ 4, treadmill capable (61.1 ± 8.9/62.9 ± 8.9)	Anodal tDCS + RAGT improved gait function with 4-week lasting effects
Saeys et al. (2014)	tDCS + MRPOT	Sham tDCS + MRPOT	1.5 mA, 20 min, 16 sessions over 4 weeks	M1	tDCS	Tinetti test; balance	First stroke (ischemic/hemorrhagic) (62.00 ± 9.61/64.53 ± 7.23)	Eight sessions of tDCS over 4 weeks improved postural control parameters

ACL, anterior cruciate ligament; AMT, active motor threshold; BBS, berg balance scale; COP, center of pressure; DBT, dynamic balance task; DLPFC, dorsolateral prefrontal cortex; FAC, functional ambulation category; FMA-LE, fugl-meyer assessment lower extremity; ICE, isometric contraction exercise; iTBS, intermittent theta-burst stimulation; MMSE, mini-mental state examination; MRPOT, multidisciplinary regular physical and occupational therapy; OSI, overall stability index; PCE, plyometric compound exercises; PT, physical therapy; RAGT, robotic-assisted gait training; RRT, routine rehabilitation therapy; rTMS, repetitive transcranial magnetic stimulation; SEBT, star excursion balance test; tDCS, transcranial direct current stimulation; TiB, time in balance; TMS, transcranial magnetic stimulation; TUG, timed up and go.

### Risk of bias in studies

**Fig. 2:**
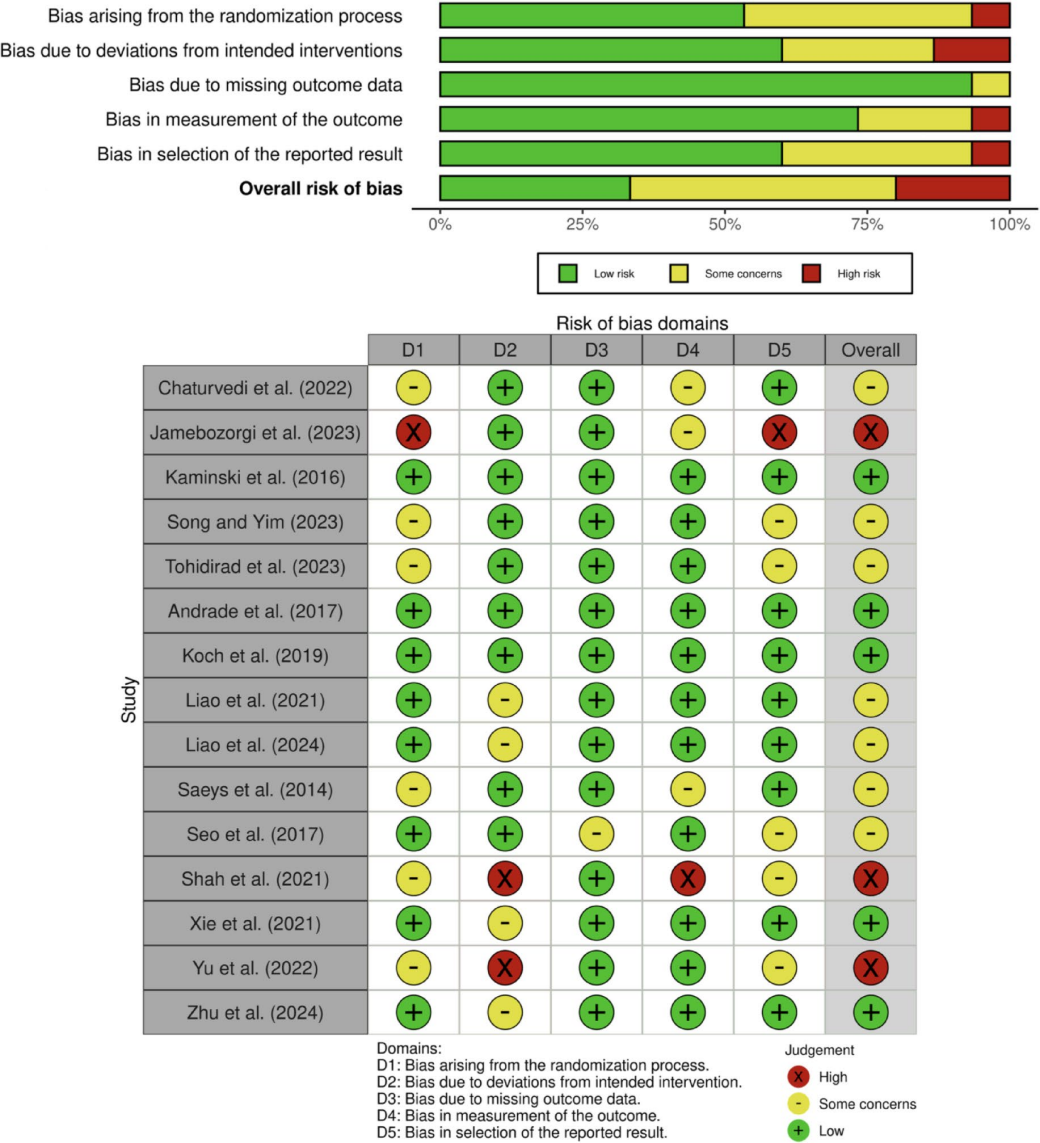
Risk of bias.

The risk of bias assessment using the RoB2 tool revealed varying quality across the included studies. Of the 15 studies evaluated, five studies (33.3%) were assessed as having an overall low risk of bias, four studies (26.7%) had some concerns, and six studies (40%) were determined to have a high risk of bias (Fig. 2).

In examining specific domains, Domain 1 (randomization process) showed that eight studies (53.3%) had low risk, while seven studies (46.7%) demonstrated some concerns. For Domain 2 (deviations from intended interventions), eleven studies (73.3%) were assessed as low risk, two studies (13.3%) had some concerns, and two studies (13.3%) showed high risk. Domain 3 (missing outcome data) displayed strong consistency, with fourteen studies (93.3%) showing low risk and only one study (6.7%) having some concerns. Domain 4 (measurement of outcome) revealed that eleven studies (73.3%) had low risk, two studies (13.3%) had some concerns, and two studies (13.3%) showed high risk. Finally, Domain 5 (selection of reported results) indicated that eight studies (53.3%) had low risk, while seven studies (46.7%) showed some concerns (Fig. 2).

### Results of syntheses

**Fig. 3:**
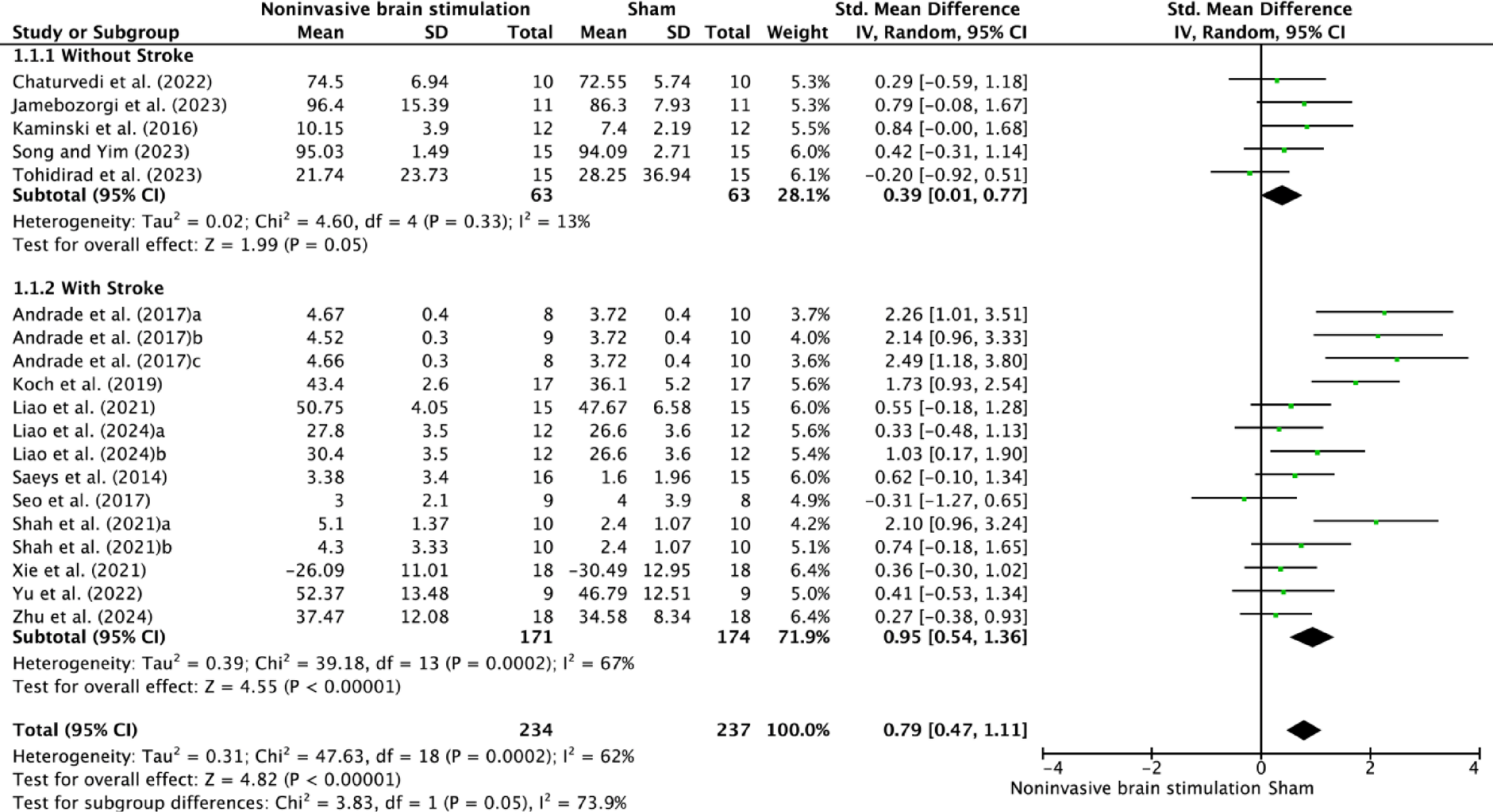
Forest plot of NIBS effects on postural control in participants with and without stroke.

The meta-analysis revealed significant overall effects of NIBS on postural control across all included studies (SMD = 0.79, 95% CI 0.47–1.11, *p* < 0.00001). Analysis of population-specific effects demonstrated distinct patterns between stroke and non-stroke groups (Fig. 3). In participants without stroke, NIBS showed a moderate effect (SMD = 0.39, 95% CI 0.01–0.77, *p* = 0.05) with low heterogeneity (I^2^ = 13%). In contrast, stroke participants exhibited substantially larger effects (SMD = 0.95, 95% CI 0.54–1.36, *p* < 0.00001) with considerable heterogeneity (I^2^ = 67%).

**Fig. 4:**
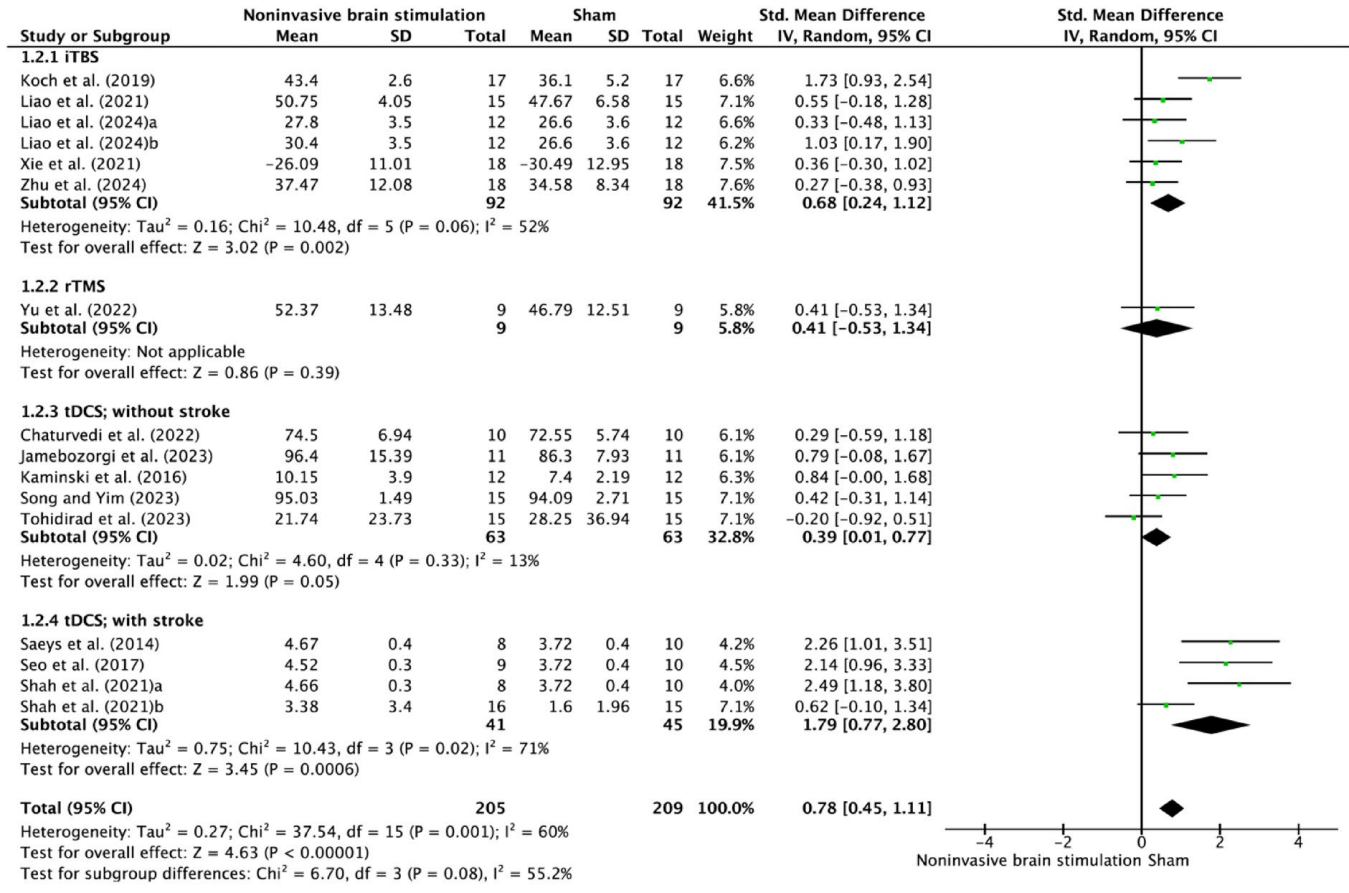
Forest plot of NIBS effects on postural control according to NIBS type.

Subgroup analysis by NIBS type (Fig. 4) revealed differential effects across modalities. iTBS demonstrated significant efficacy (SMD = 0.68, 95% CI 0.24–1.12, *p* = 0.002) with moderate heterogeneity (I^2^ = 52%). Single rTMS studies showed comparable effects (SMD = 0.41, 95% CI − 0.53–1.34), though statistical power was limited by sample size. tDCS applications demonstrated varying effects between populations: non-stroke participants showed moderate improvement (SMD = 0.39, 95% CI 0.01–0.77, I^2^ = 13%), while stroke participants exhibited more substantial gains (SMD = 1.79, 95% CI 0.77–2.80, I^2^ = 71%).

**Fig. 5:**
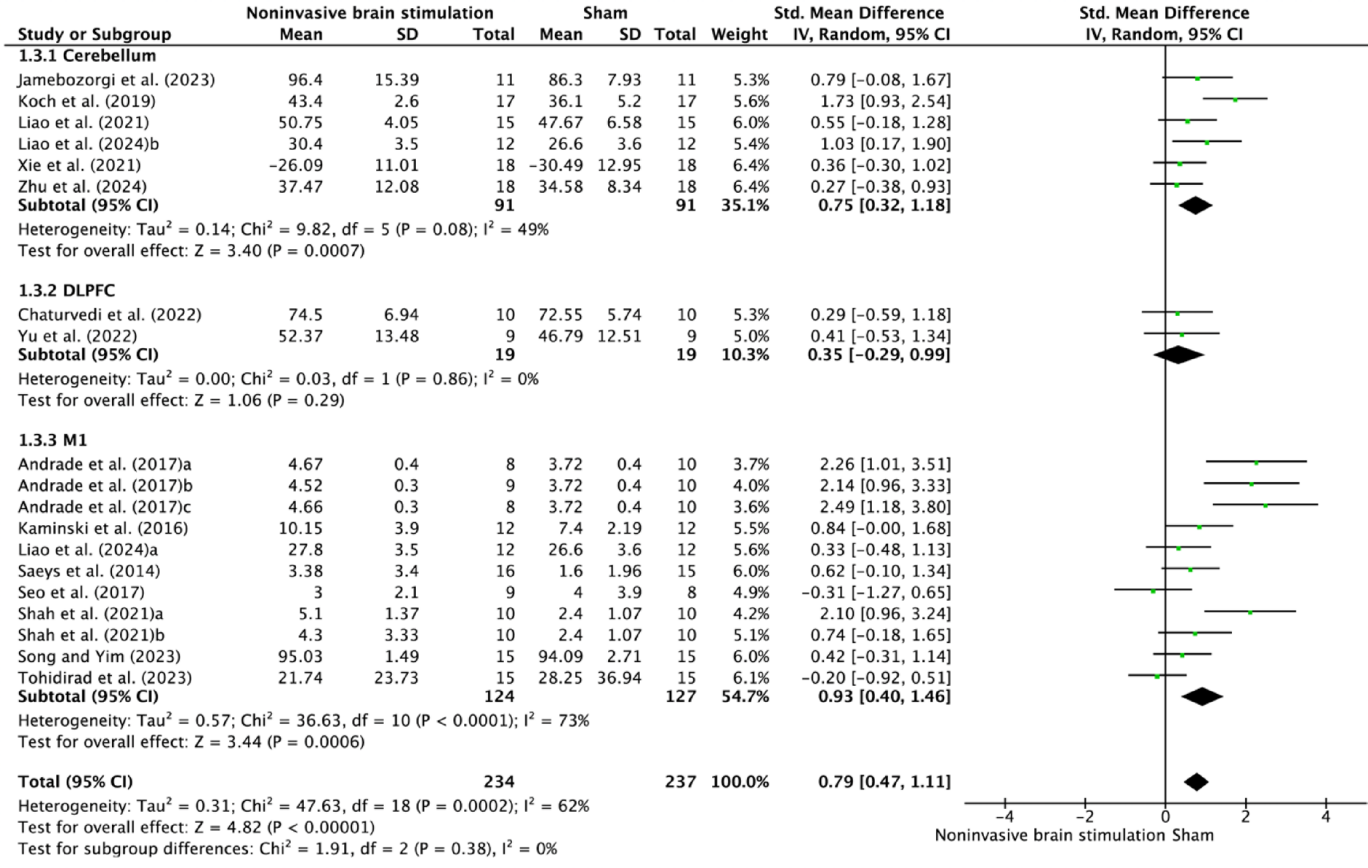
Forest plot of NIBS effects on postural control according to stimulation regions of interest (ROIs).

Analysis by stimulation region of interest (Fig. 5) indicated strongest effects for M1 stimulation (SMD = 0.93, 95% CI 0.40–1.46, *p* = 0.0006), followed by cerebellar stimulation (SMD = 0.75, 95% CI 0.32–1.18, *p* = 0.0007). DLPFC stimulation showed more modest effects (SMD = 0.35, 95% CI − 0.20–0.99, *p* = 0.29). Heterogeneity varied considerably across regions: M1 (I^2^ = 73%), cerebellum (I^2^ = 49%), and DLPFC (I^2^ = 0%).

**Fig. 6:**
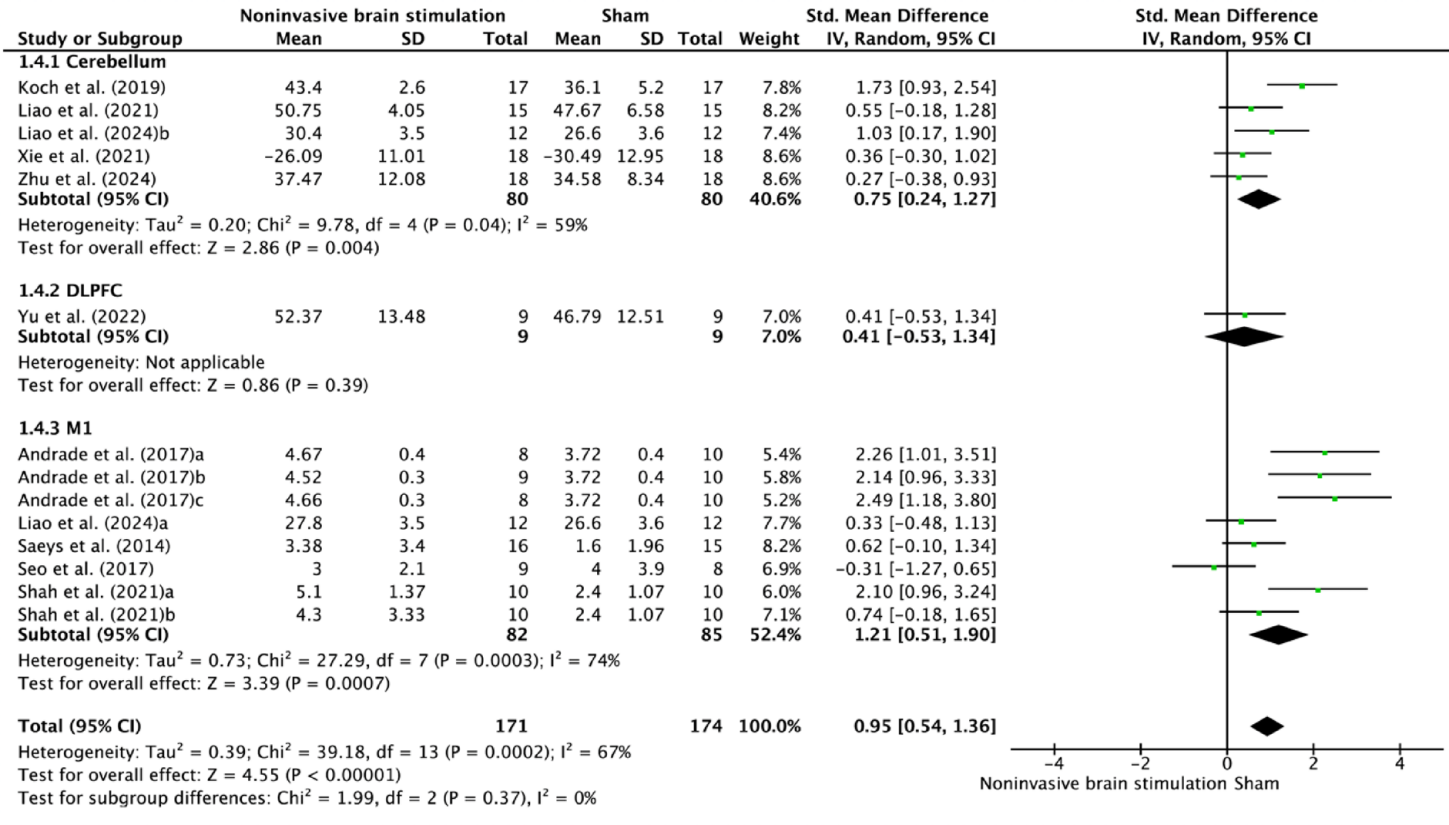
Forest plot of NIBS effects on postural control according to stimulation sites in participants with stroke.

In stroke-specific analyses (Fig. 6), cerebellar stimulation demonstrated consistent benefits (SMD = 0.75, 95% CI 0.24–1.27, *p* = 0.004) with moderate heterogeneity (I^2^ = 59%). DLPFC stimulation showed modest effects (SMD = 0.41, 95% CI − 0.53–1.34), while M1 stimulation exhibited the largest effect size (SMD = 1.21, 95% CI 0.51–1.90, *p* = 0.0007) but with substantial heterogeneity (I^2^ = 74%).

**Fig. 7:**
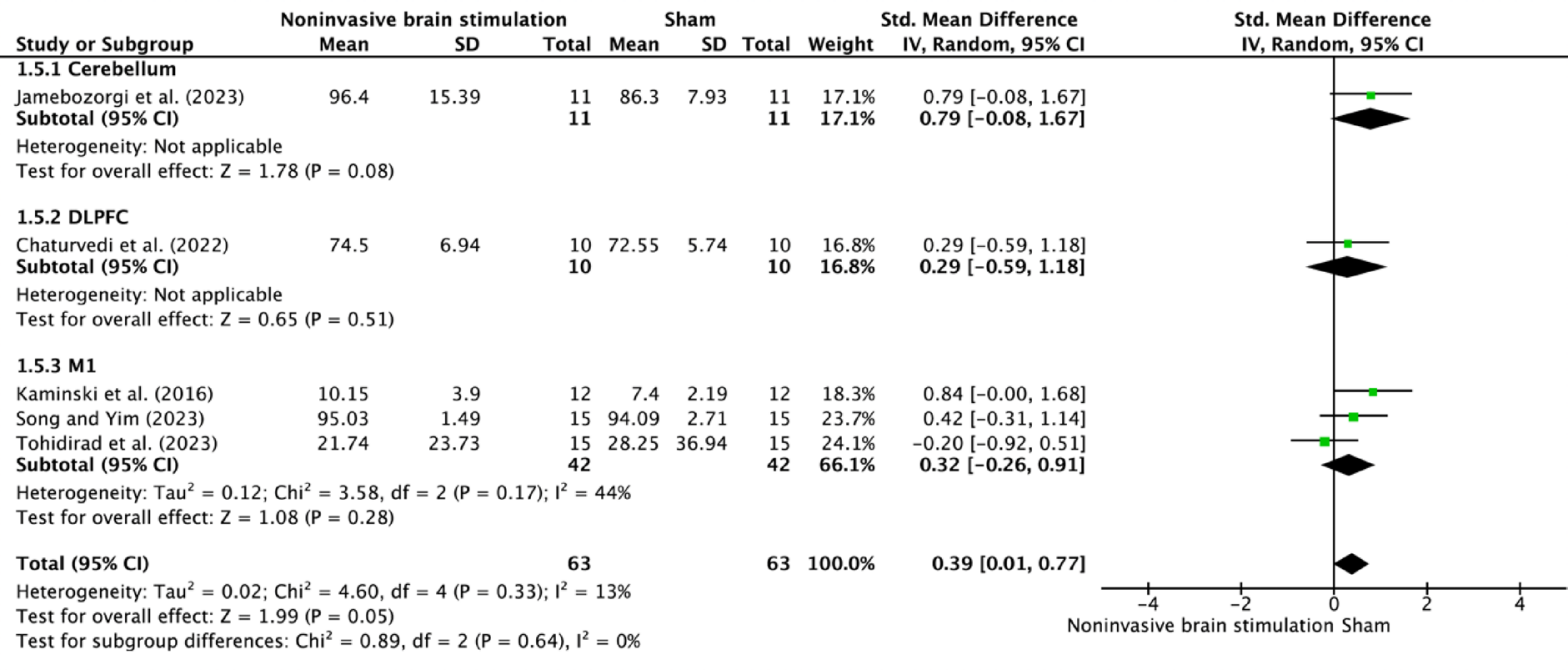
Forest plot of NIBS effects on postural control according to stimulation sites in participants without stroke.

For non-stroke populations (Fig. 7), subgroup analysis revealed comparable efficacy across stimulation sites, with cerebellar (SMD = 0.79, 95% CI − 0.08–1.67) and M1 stimulation (SMD = 0.32, 95% CI − 0.26–0.91) showing moderate effects. Notably, heterogeneity was minimal in this population (I^2^ = 13% overall), suggesting more consistent responses to NIBS interventions.

### Publication bias

**Fig. 8:**
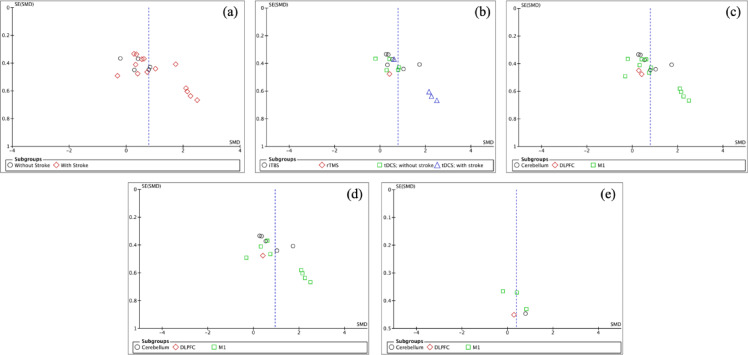
Funnel plots assessing publication bias. (**a**) Effects of NIBS on postural control in participants with and without stroke; (**b**) Effects according to NIBS type; (**c**) Effects according to stimulation ROIs; (**d**) Effects according to stimulation sites in stroke participants; (**e**) Effects according to stimulation sites in non-stroke participants.

Visual inspection of funnel plots (Fig. 8) across multiple analyses revealed generally symmetrical distributions, suggesting minimal publication bias. The primary analysis comparing stroke and non-stroke populations (Fig. 8a) demonstrated balanced scatter around the mean effect size, with studies of varying precision distributed evenly across both positive and negative effect directions. Minor asymmetry was noted in the lower precision region, potentially indicating some bias in smaller studies.

Analysis by NIBS type (Fig. 8b) showed largely symmetrical distribution for tDCS studies, while iTBS studies exhibited slight right-skewed asymmetry. This pattern suggests possible selective reporting of positive outcomes in iTBS interventions, though the limited number of studies (n < 10) necessitates cautious interpretation. The rTMS subgroup contained insufficient studies for meaningful funnel plot analysis.

Examination by stimulation regions of interest (Fig. 8c) revealed balanced distribution for M1 and cerebellar stimulation studies. DLPFC studies showed clustering around the mean effect size, though the small number of studies limits definitive conclusions about publication bias in this subgroup.

Stroke-specific analysis (Fig. 8d) demonstrated reasonable symmetry despite some scatter in lower-precision studies. The distribution pattern suggests that both positive and negative findings were represented in the literature, with no strong evidence of systematic publication bias. The non-stroke analysis (Fig. 8e) showed more limited scatter, reflecting the smaller number of studies but maintaining relative symmetry.

## Discussion

Our meta-analysis underscores a fundamental distinction in the efficacy of NIBS between stroke survivors (SMD = 0.95) and neurologically intact individuals (SMD = 0.39), highlighting a critical divergence in the mechanisms underlying neural recovery and performance enhancement. This disparity reflects fundamentally different neurophysiological states and response mechanisms. In stroke survivors, the higher effect size indicates substantial neuroplastic potential within disrupted neural circuits that can be harnessed through neuromodulation. Conversely, the more modest effect size in neurologically intact individuals suggests engagement of optimization mechanisms within already efficient neural networks. These contrasting effect magnitudes are not merely quantitative differences but represent qualitatively distinct neurobiological processes: recovery-oriented plasticity in damaged circuits versus performance enhancement in intact systems. This mechanistic distinction emphasizes the intricate interplay between baseline brain state and neuromodulatory effects, offering valuable insights for targeted therapeutic applications.

The enhanced response to NIBS observed in stroke survivors is likely attributed to the unique neurophysiological characteristics of the post-stroke brain. Unlike neurologically intact individuals, who possess optimized neural networks with limited capacity for further enhancement, stroke survivors exhibit disrupted circuits that provide a greater opportunity for reorganization^32^. This "recovery potential gradient" is evident in the higher effect sizes associated with M1 stimulation in stroke patients (SMD = 1.21)^33^. Furthermore, the substantial heterogeneity in this population (I^2^ = 67%) reflects the variability in recovery trajectories influenced by factors such as lesion location, time post-stroke, and residual neural function^34^. The superior efficacy of tDCS in stroke rehabilitation (SMD = 1.79) suggests that sustained modulation of membrane potentials offers distinct advantages over phasic stimulation protocols^35^. This finding aligns with evidence demonstrating that prolonged subthreshold stimulation facilitates synaptic plasticity and promotes reorganization at the network level.^36^. Notably, the consistency of this effect across diverse stroke populations indicates a robust mechanism underlying recovery-oriented neuroplasticity^37^.

The anatomical specificity of NIBS effects reveals a hierarchical organization of motor recovery networks. M1 stimulation demonstrated superior outcomes in stroke patients (SMD = 1.21, I^2^ = 74%), highlighting its critical role in reestablishing cortical motor commands and enhancing interhemispheric balance^38,39^. Cerebellar stimulation showed moderate yet consistent effects (SMD = 0.75, I^2^ = 49%), underscoring its complementary function in adaptive motor learning, error correction, postural control, and predictive motor adjustments^40–42^. While DLPFC stimulation demonstrated modest effects (SMD = 0.35–0.41, I^2^ = 29%), its role in supporting executive functions through top-down control appears secondary to the direct motor recovery driven by M1 and cerebellar stimulation^43–45^. This differential response pattern across regions suggests NIBS influences broader neural networks beyond local excitability changes, with effective motor recovery involving coordinated restoration of direct motor output and adaptive control mechanisms^46^. These findings highlight the potential for multisite stimulation protocols that exploit complementary network dynamics, such as combining M1 and cerebellar stimulation for enhanced outcomes^47^.

The significant heterogeneity observed in our analyses, particularly in stroke populations (I^2^ = 67%), warrants careful consideration of additional factors not directly tested in our meta-analysis. While our study design did not specifically examine timing post-stroke as a moderating variable, the literature suggests that NIBS efficacy may vary according to recovery stage, with different neural mechanisms predominating at different temporal windows.^48^ Future research should systematically investigate how the time since stroke onset influences NIBS responsiveness, potentially identifying optimal intervention windows for maximizing recovery. Similarly, the modest but consistent effects in neurologically intact individuals (SMD = 0.39, I^2^ = 13%) point to the need for more refined understanding of how NIBS protocols can be optimized for performance enhancement in already efficient neural systems^49^. This finding has practical implications for optimizing stimulation protocols for healthy individuals, potentially requiring distinct parameter configurations compared to stroke populations^50^.

The significant heterogeneity observed in stroke populations (I^2^ = 67%) underscores the influence of individual factors, including lesion characteristics, recovery stage, and baseline functional status^51^. For instance, chronic stroke patients receiving M1 stimulation exhibited notably higher effect sizes, suggesting the potential for NIBS to overcome rehabilitation plateaus by reengaging dormant neuroplastic mechanisms^8^. In contrast, the consistent yet modest effects in healthy individuals (SMD = 0.39, I^2^ = 13%) support the development of standardized protocols for performance enhancement and injury prevention. The low variability in this population facilitates the implementation of reproducible interventions tailored to specific goals, such as optimizing sports performance or reducing fall risk^52^.

Advances in stimulation technology and protocol design emphasize the need for precise parameter selection tailored to population-specific needs. The variability in efficacy across NIBS modalities documented in our analysis highlights the importance of individualized approaches^53^. For example, our finding that iTBS demonstrated significant efficacy (SMD = 0.68, I^2^ = 52%) supports its potential role in rehabilitation protocols, though the moderate heterogeneity suggests that its effects may be influenced by various factors not directly explored in our analysis^54^. Limitations of this study include the predominance of short-term follow-up periods and heterogeneity in outcome measures, which complicates cross-study comparisons. Future research should prioritize longitudinal investigations to assess the durability of NIBS-induced improvements and explore how different stimulation parameters may influence both immediate and cumulative outcomes.

This meta-analysis demonstrates distinct mechanisms underlying NIBS efficacy across populations, emphasizing the fundamental role of neural state and recovery potential. These findings provide a robust framework for advancing targeted and effective therapeutic applications in rehabilitation and performance optimization.

## Methods

### Protocol registration

This systematic review and meta-analysis was conducted following the Preferred Reporting Items for Systematic Reviews and Meta-Analyses (PRISMA) 2020 guidelines, with the protocol prospectively registered in PROSPERO (No. CRD42025634525).

### Search strategy

A comprehensive literature search was performed in October 2024 by researchers experienced in conducting meta-analyses. Independent systematic searches were executed across multiple electronic databases, including CINAHL, Embase, MEDLINE, and Web of Science, with additional sources identified through Google Scholar. The search strategy was developed based on predefined criteria to identify relevant studies examining non-invasive brain stimulation interventions for postural control in both stroke patients and healthy adults.

The search formula combined these terms as follows: (randomized controlled trial OR crossover study) AND (stroke OR brain infarction OR cerebrovascular disease OR cerebrovascular accident OR normal human OR healthy volunteers) AND (transcranial direct current stimulation OR transcranial magnetic stimulation OR repetitive transcranial magnetic stimulation OR transcranial alternating current stimulation OR paired associative stimulation OR theta burst stimulation) AND (posture OR balance OR postural balance). To ensure comprehensive yet focused results, search filters were applied to restrict studies to those published between 2014 and 2024, written in English, involving human participants aged 18–64 years, and published as peer-reviewed articles.

### Eligibility criteria

The eligibility assessment encompassed randomized controlled trials and crossover studies investigating the effects of non-invasive brain stimulation techniques, including transcranial direct current stimulation, transcranial magnetic stimulation, repetitive transcranial magnetic stimulation, transcranial alternating current stimulation, paired associative stimulation, and theta burst stimulation. Studies were considered eligible if they included either stroke patients diagnosed with brain infarction or cerebrovascular disease, or healthy adults without neurological conditions. The control conditions comprised sham stimulation, no intervention, or standard care protocols.

### Study selection

The study selection process was conducted independently by two researchers. Following the initial database searches, all identified records were exported to Microsoft Excel for systematic management. After removing duplicates, the remaining studies underwent title and abstract screening, followed by full-text assessment against the predefined eligibility criteria. Any disagreements between reviewers were resolved through discussion and consensus, with unresolved disputes adjudicated by a third reviewer.

### Data extraction

Data extraction was performed using a standardized form developed specifically for this review. The extracted information encompassed detailed study characteristics, participant demographics, intervention protocols including stimulation parameters, outcome measures, and quantitative results. Particular attention was paid to extracting data related to postural control outcomes, including static and dynamic balance parameters, postural sway measurements, and standardized balance assessments.

### Quality assessment

The methodological quality assessment employed the updated Risk of Bias 2 (RoB 2) tool for randomized controlled trials, evaluating five domains: randomization process, deviations from intended interventions, missing outcome data, outcome measurement, and selection of reported results^55^. For crossover studies, the specialized RoB 2 tool for crossover trials was utilized, incorporating additional considerations specific to the crossover design, including carryover effects and washout period adequacy^56^. Two independent researchers conducted the quality assessment, with discrepancies resolved through discussion and consensus.

### Statistical analysis

Statistical analyses were conducted using RevMan (Review Manager, version 5.4, The Cochrane Collaboration, Copenhagen, 2020). Effect sizes were calculated using standardized mean differences with 95% confidence intervals. A random-effects model was employed to account for anticipated heterogeneity among studies^57^. The assessment of heterogeneity utilized both the chi-square test and I^2^ statistic, with I^2^ values above 75% indicating high heterogeneity and values below 40% suggesting low heterogeneity^58^. To evaluate potential publication bias across the included studies, visual inspection of funnel plot asymmetry was planned^59^. However, acknowledging the limitations of funnel plot analysis with small sample sizes, this assessment would only be conducted if ten or more studies were available for analysis, as fewer studies would not provide sufficient power to distinguish chance from real asymmetry^60^. Where appropriate, subgroup analyses were planned to explore the differential effects of various NIBS modalities and specific outcome measure categories across stroke and healthy populations.

## Data Availability

All data generated or analysed during this study are available to readers upon request to the corresponding authors, Sangha Cha (ldoavney@gmail.com) and Kang Hee Cho (khcho@cnu.ac.kr).
